# Supplementary material for: Spatial variation and factors associated with home delivery after ANC visit in Ethiopia; spatial and multilevel analysis
Source: PLoS One. 2022 Aug 25;17(8):e0272849. doi: 10.1371/journal.pone.0272849 (PMC9409554; doi:10.1371/journal.pone.0272849)
Supplement: Attachment — Submitted filename: Response to Reviewers.docx [file pone.0272849.s001.docx]

**Rebuttal letter	Date April 20,2022**

**Subject; submission of revised manuscript (PONE-D-21-33331)**

**SPATIAL VARIATION AND FACTORS ASSOCIATED WITH HOME DELIVERY AFTER ANC VISIT IN ETHIOPIA; SPATIAL AND MULTILEVEL ANALYSIS**

**Hiwotie Getaneh Ayalew**

**To PLOS ONE**

Dear all,

We would like to thank you for these constructive, building, and improvable comments on this manuscript that would improve the substance and content of the manuscript. We considered each comment and clarification question of editors and reviewers on the manuscript thoroughly. Our point-by-point responses for each comment and question are described in detail on the following pages. Further, the details of changes were shown by track changes in the supplementary document attached. The manuscript language was further improved in the revised manuscript. and we follow journal guidelines. I have attached recent comments in a point-by-point response.

**Version 1; editor’s comments**

1. Please ensure that your manuscript meets PLOS ONE's style requirements, including those for file naming.

**Authors’ response**; thank you dear editor we have prepared the documents based on PLOS ONE requirements.

2. Thank you for stating the following in your Competing Interests section.

**Authors’ response;** thanks, dear editor.

3. Thank you for submitting the above manuscript to PLOS ONE. During our internal evaluation of the manuscript, we found significant text overlap between your submission and the following previously published work.

-<https://www.researchsquare.com/article/rs-55326/v1>

**Authors’ response;** Thank you, dear editor, we have included the reference and we have rephrased the duplicated texts and words in the revised manuscript.

**Version 2; reviewers’ comments**

1. Is the manuscript technically sound, and do the data support the conclusions?
The manuscript must describe a technically sound piece of scientific research with data that supports the conclusions. Experiments must have been conducted rigorously, with appropriate controls, replication, and sample sizes. The conclusions must be drawn appropriately based on the data presented.

Reviewer #1: Yes

Reviewer #2: Yes

**Authors’ response**; thank you, dear reviewers

2. Has the statistical analysis been performed appropriately and rigorously?

Reviewer #1: Yes

Reviewer #2: No

**Authors’ response**; thank you, dear reviewers. We have improved the revised version of the manuscript.

3. Have the authors made all data underlying the findings in their manuscript fully available?
The [PLOS Data policy](http://www.plosone.org/static/policies.action#sharing) requires authors to make all data underlying the findings described in their manuscript fully available without restriction, with rare exception (please refer to the Data Availability Statement in the manuscript PDF file). The data should be provided as part of the manuscript or its supporting information, or deposited to a public repository. For example, in addition to summary statistics, the data points behind means, medians and variance measures should be available. If there are restrictions on publicly sharing data—e.g. participant privacy or use of data from a third party—those must be specified.

Reviewer #1: No

Reviewer #2: Yes

**Authors’ response**; Thank you, dear reviewers. We have stated that the data was fully available without restriction in the revised manuscript at the declaration session.

4. Is the manuscript presented in an intelligible fashion and written in standard English?
PLOS ONE does not copyedit accepted manuscripts, so the language in submitted articles must be clear, correct, and unambiguous. Any typographical or grammatical errors should be corrected at revision, so please note any specific errors here.

Reviewer #1: No

Reviewer #2: No

**Authors’ response**; thanks a lot dear reviewer. we have critically improved the readability of the manuscript. Please see the revised version.

**Reviewer 1 Comments**

After having carefully read the manuscript and taking notes on overall strengths and weaknesses, I share my comments below.
**General comments** **and suggestions 1;**  I am happy and glad to the editor for sending me the paper for reviewing.I am happy reading the version where authors addressed an important topic, however I have some observations. Please re-edit the conclusion of abstract. Is is liking odd to read. It is better.. starting of two sentences. It is large and the repetition of the conclusion of the main manuscript. I wonder authors are working with Ethiopia data but in the conclusion they wrote “It is better to give special attention for women who lived in Somalia, Amhara, Afar, Oromia, Benishangul Gumuz, Tigray and SNNP regions” do you have any clarification for this?

**Authors’ response;** thanks a lot dear reviewer for your critical comment to improve the manuscript. we have fully accepted your comment we have to avoid and reduce the repetition of the conclusion of the abstract.

These regions Somalia, Amhara, Afar, Oromia, Benishangul Gumuz, Tigray and SNNP regions were detected when we conduct spatial analysis. we find hotspot regions in hot spot analysis and high-risk areas of home delivery after ANC visit with spatial scan statistics scanning window in these regions. Therefore based on the finding it is important to recommend those regions. We kindly request to see the revised manuscript at the spatial analysis result session.

**Comment 2;** In the introduction section I didn’t found the strong rationality of the study, that is completely missing. I would suggest to re-work on this to establish a strong rationality for the work.

**Authors’ response**; thanks a lot dear reviewer for your unlimited effort to improve the manuscript. We accepted your comment and included sound justification in the revised manuscript.
**Comment 3**; In the first line of the introduction they told “skilled health care providers” I am fear for this line. Would it be “skilled birth attendants” please recheck.

**Authors’ response;** thanks, dear reviewer for your detailed revision. We have accepted the comment and we correct the revised manuscript.

**Comment 4;** Studies have shown that mothers who have ANC visits are more likely to give birth at health institutions” references missing from this line.

**Authors’ response;** Thanks a lot dear reviewer. We have added the reference in the revised manuscript.

**Comment 5;** I think the Study setting is irrelevant, If I were you I would delete this section. Authors used in some line “unskilled personal” or “unskilled personnel” please solve this issue.

**Authors’ response;** thank you in advance dear reviewer. We have delated the study setting and we also have taken an edition on the issue of “unskilled personal” in the revised manuscript. **Comment 6;** “being in the poor” or “being poor”. First two lines of the discussion is unnecessary.

**Authors’ response;** thank you dear reviewer we have written the interpretation of the variable wealth index as “being in the poor” and we have also removed the first line of the discussion session in the revised manuscript.

**Comment 7**; “Possibly it might be most poor women in Ethiopia live in rural areas, in the absence of transportation and long-distance travel may lead to home birth.” References missing for this line

**Authors’ response**; thanks dear reviewer we have to appreciate your comments. We have added some references and we kindly request you to see them in the revised manuscript.

**Comment 8**; Need “compared to urban women This finding.” full stop or anything else.
“This may also be due to distance to the health facilities, ambulance delays, inaccessibility of information, and low educational status of women from rural areas” does it means rural areas of Ethiopia are underprivileged, please extend this line for further clarification.

**Authors’ response**; thanks a lot dear reviewer for your deep comments. We have added a full stop at the end of “compared to urban women” Additionally for the next comment we have further extended our clarification for the explanation. Since it does not mean that rural women are underprivileged rather it means due to the topographic difficulty of the country and scattered living distribution of households and health institutions, there is long distance and difficult roads so this leads to home delivery. We also added a reference for this comment in the revised manuscript.

**Comment 9;** The possible explanation might be due to scarcity of resources and poor clinical settings, in which most of the services are concentrated in urban areas or city administrates “references missing.

**Authors’ response;** thank you, dear reviewer. We have added a reference and we kindly asked you to see the revised manuscript.

**Comment10;** In addition, those most agrarian women’s are farmers mostly had sudden labor onset and short duration which… I really fear of this line. Do you have any evidence or supporting literature?

**Authors’ response;** thanks dear reviewer for your constructive comments. We have added evidence of supporting kinds of literature in the revised manuscript. We kindly ask you to see the revised manuscript.

**Reviewer #2 comments**

**Comment** 1. Why the marital status (married/unmarried) variable included in individual level variables.

**Authors’ response;** thanks dear reviewer we accepted your comment. The analytic model of this study was the multilevel model and it includes individual and community level factors.so factors that can be directly found in the data set for each study participants were considered as individual-level factors like marital status. Whereas community-level factors were generated by aggregating individual-level factors.

**Comment 2;** why wealth status is coded in 3 categories? The DHS data has 5 categories.

**Authors’ response;** thank your dear reviewer for your comment**.** DHS has five categories for wealth index, but for our analysis, we have recoded wealth index into three categories, due to that it did not fulfill the chi-square assumption as we used it as five categories so we changed it to three categories. Some kinds of literature also use wealth index as three categories for analysis
**comment 3;** Media exposure can be coded as no, partial, full

**Authors’ response;** thank you, dear reviewer. we have used media exposure for analysis as exposed (yes)and unexposed (no) for media.

. **Comment 4;** what does it mean birth interval (short/long)?

**Authors’ response;** thank you, dear reviewer. We strongly appreciate your comment. Birth interval is the duration which refers to the time interval from delivery to the next subsequent pregnancy of the fetus. It is classified as short if it is less than 15 months and longer if it is greater than or equal to 15 months and it also has an effect on place of delivery as delivered in Textbook of Williams Obstetrics 25th edition.

**Comment 5**; why both the residency and region variables included in the models? It could lead to the problem of multicollinearity.

**Authors’ response;** Thanks a lot dear reviewer for your interesting and constructive comments. We initially checked the VIF (variance inflation factor) of all individual and community level variables before fitting the model and there was no multicollinearity in between variables.
**comment 6**; Home delivery after ANC visit was spatially clustered in Ethiopia. What is the role of independent variables in this phenomenon?

**Authors’ response;** thanks dear reviewer we appreciate your comments. In this study, we used two models spatial and multilevel analysis. In the case of spatial analysis, we only used the GPS data set and the outcome variable to identify high-risk areas of home delivery after ANC visit in Ethiopia, whereas in the multilevel model to identify determinants of home delivery after ANC visit we used independent variables (individual level and community level factors). therefore independent variables for spatial analysis to determine whether home delivery was spatially clustered or not have no role.

**Comment 7**; the manuscript has many grammatical and typing errors.

**Authors’ response;** thanks a lot dear reviewer we have critically improved the grammatical error and readability of the manuscript. Please see the revised version.

**Thanks, a lot!!!**
